# Extracellular Vesicles from Bone Marrow‐Derived Mesenchymal Stem Cells Improve Survival from Lethal Hepatic Failure in Mice

**DOI:** 10.1002/sctm.16-0226

**Published:** 2017-02-18

**Authors:** Hiroaki Haga, Irene K. Yan, Kenji Takahashi, Akiko Matsuda, Tushar Patel

**Affiliations:** ^1^ Department of Transplantation, Mayo ClinicJacksonvilleFloridaUSA; ^2^ Department of Cancer BiologyMayo ClinicJacksonvilleFloridaUSA

**Keywords:** Extracellular vesicles, Tumor necrosis factor, Fulminant hepatic failure, Cytokines, Apoptosis

## Abstract

Stem cell‐based therapies have potential for treatment of liver injury by contributing to regenerative responses, through functional tissue replacement or paracrine effects. The release of extracellular vesicles (EV) from cells has been implicated in intercellular communication, and may contribute to beneficial paracrine effects of stem cell‐based therapies. Therapeutic effects of bone‐marrow derived mesenchymal stem cells (MSC) and vesicles released by these cells were examined in a lethal murine model of hepatic failure induced by d‐galactosamine/tumor necrosis factor‐α (TNF‐α). Systemically administered EV derived from MSC accumulated within the injured liver following systemic administration, reduced hepatic injury, and modulated cytokine expression. Moreover, survival was dramatically increased by EV derived from either murine or human MSC. Similar results were observed with the use of cryopreserved mMSC‐EV after 3 months. Y‐RNA‐1 was identified as a highly enriched noncoding RNA within hMSC‐EV compared to cells of origin. Moreover, siRNA mediated knockdown of Y‐RNA‐1 reduced the protective effects of MSC‐EV on TNF‐α/ActD‐mediated hepatocyte apoptosis in vitro. These data support a critical role for MSC‐derived EV in mediating reparative responses following hepatic injury, and provide compelling evidence to support the therapeutic use of MSC‐derived EV in fulminant hepatic failure. Stem Cells Translational Medicine *2017;6:1262–1272*

**Significance Statement**

We report a dramatic improvement in survival after the systemic administration of stem cell‐derived extracellular vesicles (EV) in a mouse model of experimental lethal hepatic injury. The beneficial effect of these vesicles on reducing mortality from lethal hepatic injury involves modulation of the inflammatory response and activation of protective mechanisms to limit cell death. We further identify Y‐RNA‐1 as highly enriched within stem cell‐derived vesicles and show a protective effect on hepatocyte apoptosis. Most importantly, these studies support the development of a novel therapeutic strategy for liver failure, based on use of stem cell‐derived EV.

## Introduction

Acute or fulminant hepatic failure is a serious condition and can be fatal. Hepatic failure can occur in the setting of liver injury with impaired functional and synthetic capacity that can result in encephalopathy or coma in patients with previous normal liver or well compensated liver disease 1, 2. Although orthotopic liver transplantation is an effective intervention, its use is limited by donor organ scarcity as well as by medical, financial, and socioeconomic considerations 3. There is a need for new therapeutic options to reduce liver injury and to enhance regeneration in order to improve the outcome of acute or fulminant hepatic failure.

Mesenchymal stem cell (MSC) transfusions can improve liver function in acute hepatic failure 4, 5, 6, 7. Moreover, bone marrow‐derived MSCs are known to exert immunoregulatory and anti‐apoptotic effects 8. Stem cells are attractive as a source of tissue replacement because of their regenerative ability and multipotent capability. Therapeutic benefits with the use of stem cells could result from replacement of functioning epithelia and tissue. However, the window of opportunity for a therapeutic benefit may be insufficient for replacement strategies that require hepatic differentiation, a process which can extend from several days to weeks. Alternatively, stem cells may have beneficial effects through paracrine effects mediated by release of soluble factors and extracellular vesicles (EV) from these cells 9, 10. Indeed, the importance of paracrine factors on liver regeneration have been recognized and systemic injection of conditioned medium (CM) from MSC has been shown to improve hepatocellular apoptosis and stimulate hepatocyte regeneration in animal models of acute hepatic failure 10, 11, 12, 13. CM is heterogeneous and can contain soluble factors such as cytokines, chemokines, and growth factors, as well as particles such as EV.

MSC, as well as many other cell types can release EV such as microvesicles and exosomes into the extracellular space 9, 14, 15, 16, 17, 18. EV can also be detected in biological fluids such as blood, urine, and ascites fluid 19. EV are involved in cell–cell communication and can transfer their content to modulate cellular activities in recipient cells. We have shown that malignant hepatocytes can transfer genetic information by release of EV that can modulate recipient cell behavior 16. Biologically active molecules contained within EV include proteins, DNA, messenger RNA, and noncoding RNA such as microRNA and long noncoding RNA (lncRNA) 17, 20. While the role of MSC‐derived soluble factors as effectors of the paracrine effect have been investigated, the contribution of MSC‐derived EV are not as well characterized. Thus, our goals were to evaluate the effects of stem cell‐derived EV on experimental hepatic failure and thereby to determine their potential utility as therapeutic agents for tissue repair and functional restoration in acute liver failure.

## Materials and Methods

### Cells and Cell Culture

Mouse bone marrow‐derived mesenchymal stem cells (mMSC) were obtained from Life Technologies (Grand Island, NY, Lifetechnologies.com) and grown in Dulbecco's modified Eagle medium/Nutrient Mixture F‐12 containing MSC‐Qualified fetal bovine serum (Gibco, Grand Island, NY, thermofisher.com). Human bone marrow‐derived mesenchymal stem cells (hMSC) were obtained as previously described 21, 22, 23, 24. Nonmalignant human hepatocytes (HH) were obtained from Sciencell (Carlsbad, CA, sciencell.com) and cultured as recommended by the supplier.

### Isolation and Characterization of EV

**Figure 1:**
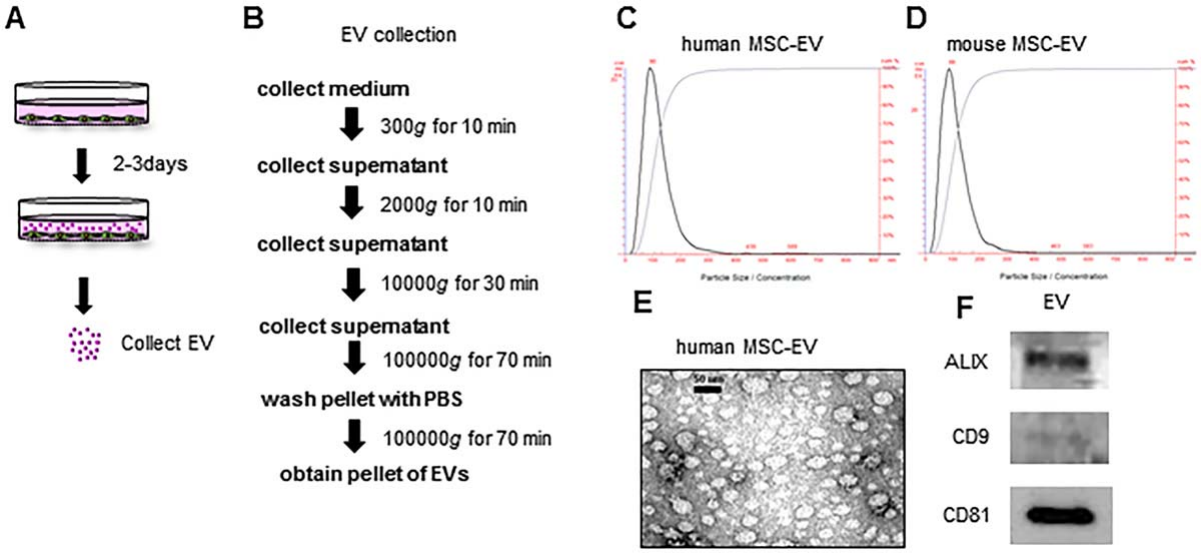
Characterization of bone marrow MSC‐derived EV. **(A, B)**: EV were isolated from conditioned medium obtained from human MSC in culture by ultracentrifugation. **(C, D)**: Particle quantitation was performed using nanoparticle tracking analysis. EV isolated from human MSC have a mean size of 116 ± 46 nm and a peak size of 92 nm (C), whereas EV isolated from mouse MSC a mean size of 112 ± 56 nm and a peak size of 88 nm (D). **(E)**: Transmission electron microscopy was performed on a whole mount of vesicles isolated from human MSC. **(F)**: Western blot analysis for selected vesicle associated markers was performed on EV isolated from human MSC. Abbreviations: EV, extracellular vesicles; MSC, mesenchymal stem cell.

Vesicle‐depleted medium was used for all studies and isolation of EV was performed as described 16, 25. EV count was quantitated using NanoSight (NanoSight Ltd., Amesbury, UK, malvern.com). Isolated EVs were resuspended in phosphate‐buffered saline (PBS) and stored at −80°C (Fig. 1A, 1B). Transmission electron microscopy was performed on whole mounts of isolated EV as previously described 24.

### Animal Studies

All studies involving animals were performed in accordance with institutional animal care and use committee approved protocols. Lethal hepatic failure was induced in 10‐ to 12‐week‐old male C57Bl/6 mice (Jackson Laboratory, Bar Harbor, ME, jax.org) by intraperitoneal (i.p.) injection of 20 milligram per body d‐galactosamine (Sigma‐Aldrich, St. Louis, MO, sigmaaldrich.com) and 0.3 microgram per body recombinant tumor necrosis factor‐α (TNF‐α; R&D systems, Minneapolis, MN, rndsystems.com) 26, 27. After d‐galactosamine/TNF‐α administration, mice received injections of stem cells (2 × 10^6^ cells per body hMSC or mMSC i.p.), stem cell‐derived EV (2 × 10^8^ to 2 × 10^10^ particles per body hMSC‐EV, or mMSC‐EV, i.p./i.v.), PBS diluent control (PBS, i.p./i.v.) or no additional treatment. Each intervention group consisted of 6 to 8 mice each. Mice were observed for 24 hours after d‐galactosamine/TNF‐α injection. Studies of cryopreserved EV were performed using mMSC‐EV that had been stored at −80°C for 1 or 3 months after isolation. For biodistribution studies, mMSC‐EV were incubated with 6 µmol/l DiR (1,1′‐dioctadecyl‐3,3,3′,3′‐tetramethylindotricarbocyanine iodide) (Life Technologies, Grand Island, NY) for 30 minutes. Labeled EV were washed with 20 ml of PBS, ultracentrifuged, and injected i.p. or i.v. 2 × 10^10^ particles per body into male C57Bl/6 mice. Organs were harvested 6 hours after EV injection, and accumulation of labeled EV was quantitated ex situ using an in vivo imaging system (IVIS) (Perkin Elmer, Waltham, MA).

### Histopathology and Serum Analyses

Liver and blood were obtained 6 hours after injection of d‐galactosamine/TNF‐α followed by i.p. injections of human or mouse MSC‐EV (2 × 10^10^ particles per body) or PBS. Blood was collected by cardiac puncture and serum was obtained by centrifugation (3,000*g*, 5 minutes). Serum biochemical analyses and quantitation of cytokines was performed using Standard Blood Chemistry Panels (IDEXX BioResearch, North Grafton, MA, idexx.com). Tissue staining for hematoxylin and eosin and immunohistochemistry was performed by the Mayo Clinic Florida Cancer Biology Histology Core Resource. Briefly, slides were deparaffinized and hydrated with distilled water, and antigen retrieval performed by soaking slides in EDTA in a 100°C steamer for 25 minutes. A protein block was prepared using Protein Block Serum‐Free (Dako, Carpinteria, CA, dako.com) and immunohistochemistry was performed using a primary antibody against cleaved Caspase‐3 rabbit polyclonal antibody (dilution 1:100) (Cell Signaling Danvers, MA) or rat anti‐mouse F4/80 monoclonal antibody (dilution 1:200) (AbD Serotec, Raleigh, NC), and EnVision Plus System‐HRP Labeled Polymer Anti‐Rabbit or Anti‐Rat secondary antibodies (Dako, Carpinteria, CA) along with 3,3‐diaminobenzidine (Dako, Carpinteria, CA). Whole tissue sections were scanned at ultraresolution on the ScanScope XT (Aperio Technologies, Vista, CA). Caspase‐3 and F4/80 positive density areas were quantitated by pixel counting using ImageScope version 12.1 (Aperio Technologies, Vista, CA).

### Western Blot Analysis

EV were lysed and Western blot analysis was performed on lysates as previously described 24 using primary antibodies: mouse monoclonal antibody Alix (1:500) (Novus Bio, Littleton, CO), mouse monoclonal antibody CD9 (1:200) (Santa Cruz, Dallas, TX, scbt.com), mouse monoclonal antibody CD81 (1:200) (Santa Cruz, Dallas, TX), with protein expression noted relative to that of Actin.

### Isolation of RNA

Total RNA was extracted from cells using Trizol (Life Technologies) or from EV using ExoQuick‐TC (System Biosciences, Mountain View, CA). Human MSC were plated in 11 ml of EV‐depleted medium on collagen‐coated 10‐cm dishes. After 3 days, the medium was collected and sequentially centrifuged at 3,000*g* for 15 minutes to remove cells and cell debris. The supernatant was transferred to a sterile vessel and combined with 2 ml ExoQuick‐TC. After an overnight precipitation at 4°C, total RNA was extracted using SeraMirTM exosome RNA amplification kit (System Biosciences). RNA concentration was measured using NanoDrop ND‐2000 (Nano‐Drop Technologies, Wilmington, DE).

### Polymerase Chain Reaction (PCR) Analysis

RNA was treated with RNase‐free DNase I (Qiagen, Valencia, CA). One microgram of RNA was reverse‐transcribed to cDNA using iScript cDNA Synthesis Kit (BIO‐RAD Laboratories, Inc., Hercules, CA), and real‐time quantitative Reverse‐Transcription (RT)‐PCR was performed using a LightCycler 96 System (Roche Diagnostics, Mannheim, Germany) to detect U6 and Y RNA‐1 using SYBR green I (SYBR Advantage qPCR Premix, Clontech, Mountain View, CA). The following PCR primers were used: human Y RNA‐1 primers, forward: 5′‐ TCCGAAGGTAGTGAGTTATCT‐3′, reverse: 5′‐GGGAAAGAGTAGAACAAGGAG‐3′, mouse Y RNA‐1 primers, forward: 5′‐TCCGAAGGTAGTGAGTTATCT‐3′, reverse: 5′‐GGGGAAAGTGTAGAACAGGAG‐3′; U6 primers, forward: 5′‐CTCGCTTCGGCAGCACA‐3′, reverse: 5′‐AACGCTTCACGAATTTGCGT‐3′. LncRNA expression analysis was performed using the LncProfiler quantitative PCR (qPCR) Array Kit (System Biosciences, Mountain View, CA, systembio.com). RNA from EV or donor cells (*n* = 4 per each cell line) were treated with DNase I and 2 μg of DNase‐treated RNA was reverse transcribed, prior to PCR using SYBR Green (2X Maxima SYBR Green with Rox, Fermentas, Glen Burnie, MD). The cycle number at which the reaction crossed a threshold (CT) was determined for each gene. Raw CT values were normalized using a U6 snRNA CT value (ΔCT = CT_lncRNA_ − CT_U6 snRNA_). The relative amount each lncRNA in EVs relative to donor cells was described using the equation 2^−ΔΔCT^ where ΔΔCT = ΔCT_EV_ − ΔCT_donor cell_.

### Transfection of siRNA

Small interfering (si)RNA against Y‐RNA‐1 was designed using siDESIGN center (Dharmacon, Lafayette, CO). siRNAs against Y‐RNA‐1 (Y‐RNA‐1 siRNA; UUAUCUCAAUUGAUUGUUCACUU) or nontargeting (NT) control siRNA (siGENOME NT siRNA) were purchased from Dharmacon, dharmacon.gelifesciences.com. Transfection of human MSC was performed using Amaxa cell line nucleofector Kit V (Lonza). Cells were suspended in Nucleofector to a final concentration of 5 × 10^5^ cells per 100 microliter, and then transfected with either 50 nM and 100 nM of Y‐RNA1‐siRNA or 2 μg Green Fluorescent Protein (GFP) plasmid using the Nucleofector II Device (Lonza). After electroporation, cells were recovered by adding 500 µl media without antibiotics into the cuvette and then plated onto a six‐well cell culture plate. After 6 hours, the media was replaced with media containing antibiotics.

### Caspase 3/7 Activity Assay

Cells were plated (2 × 10^4^ per well) in 96‐well collagen‐coated plates for 6 hours prior to incubation with or without hMSC‐EV or EV from hMSC transfected with siRNA (Y‐RNA‐1 or NT control) for 24 hours. Cells were then pretreated with actinomycin D (ActD) 200 ng/ml for 30 minutes, prior to incubation with TNF‐α 20 ng/ml. Caspase‐3/7 activity was assessed after 12 hours using Caspase‐GloR3/7 Assay kit (Promega, Madison, WI, promega.com) and the FLUOstar Omega Microplate Reader (BMG Labtech, Cary, NC). Data were expressed as relative luminescence values relative to those of controls.

### Statistical Analysis

Data were expressed as the mean and standard error from at least three replicates. Comparisons between groups were performed using the two‐tailed Student's *t* test. For survival experiments, a log‐rank test was conducted. Results were considered to be statistically significant when *p* < .05.

## Results

### Characterization of Bone Marrow MSC‐Derived EV

We first characterized EV released by human bone marrow‐derived mesenchymal stem cells (hMSC) in vitro. Vesicles were isolated using an ultracentrifugation‐based protocol and separated at a density of 1.12 g/ml on density gradient centrifugation (Fig. 1A, 1B). A homogeneous population of hMSC‐derived extracellular vesicles (hMSC‐EV) are released into culture supernatant from cell cultures in vitro. Isolated vesicles were spherical membrane‐bound vesicles (on electron microscopy) with a mean diameter of 116 ± 46 nm (by nanoparticle tracking analysis) (Fig. 1C, 1E), and expressed ALIX, CD9, and CD81 (on immunoblot analysis) (Fig. 1F). While the morphological and biochemical features are consistent with those of exosomes, a specific subset of vesicles that have a distinctive biogenesis, we refer to these as EV because their biogenesis has not been directly ascertained. A similar protocol was used for the isolation and characterization of murine mesenchymal stem cell‐derived EV (mMSC‐EV), which resulted in a population of vesicles with a mean diameter of 112 + 56 nm (Fig. 1D).

### Enhanced Hepatic Uptake of MSC‐EV Following d‐Galactosamine/TNF‐α Induced Liver Injury

**Figure 2:**
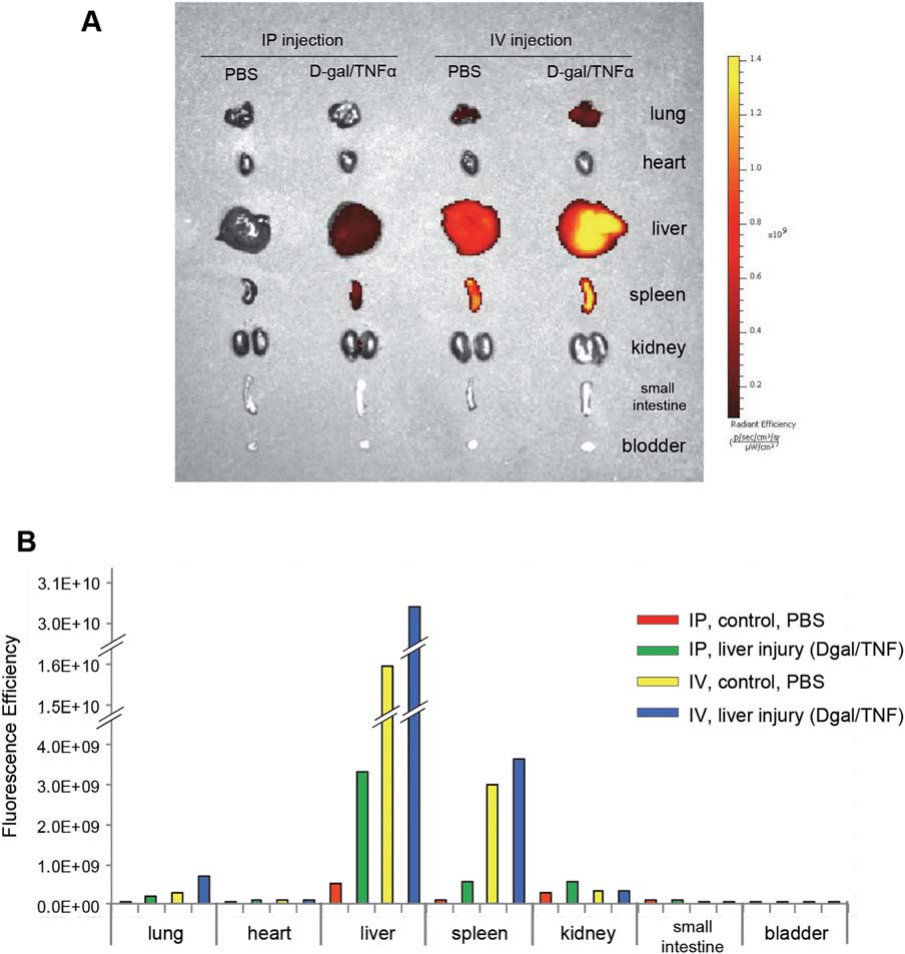
Biodistribution of labeled murine mesenchymal stem cell‐derived extracellular vesicles (mMSC‐EV). DiR‐labeled mMSC‐EV were administrated intraperitoneally or intravenously to mice following administration of either 20 mg d‐galactosamine/0.3 μg TNF‐α to induce hepatic injury, or PBS (control). **(A)**: Tissue accumulation of labeled EV was detected ex vivo after 6 hours, and (B) the intensity of fluorescent signal was measured using an IVIS. EV uptake in the liver is increased following TNF/d‐galactosamine induced hepatic injury compared to controls. Abbreviations: PBS, phosphate‐buffered saline; TNF, tumor necrosis factor; TNF‐α, tumor necrosis factor‐α.

To evaluate the contribution of MSC‐derived EV to regenerative responses following liver injury, we used a murine model of lethal hepatic failure. The coadministration of TNF‐α and d‐galactosamine results in hepatic failure, characterized by tremulousness, an impaired ability to walk straight or to recover from a supine position, loss of eyelash reflex and eventual coma. We observed a mortality rate in C57Bl/6 mice of 100% after 24 hours of receiving TNF‐α (0.3 microgram per body) and d‐galactosamine (20 microgram per body) alone (*n* = 3), or with concomitant i.p. administration of PBS (*n* = 7). To examine the biodistribution of mMSV‐EV following hepatic injury, mMSC‐EV were labeled with the near infrared fluorescent dye DiR and administered intraperitoneally or intravenously in mice receiving d‐galactosamine/TNF‐α to induce hepatic injury or PBS (controls). Organs were harvested after 6 hours for ex vivo imaging. Hepatic uptake of EV was increased in mice receiving d‐galactosamine/TNF‐α compared to controls, in which very little uptake was detected within the liver (Fig. 2). Increased hepatic uptake was noted with either i.p. or intravenous administration. These data show enhanced uptake of MSC‐EV within an injured liver and support the use of systemic administration of MSC‐EV to target the injured liver.

### MSC‐EV Improve Survival in a Mouse Model of Fulminant Hepatic Failure

**Figure 3:**
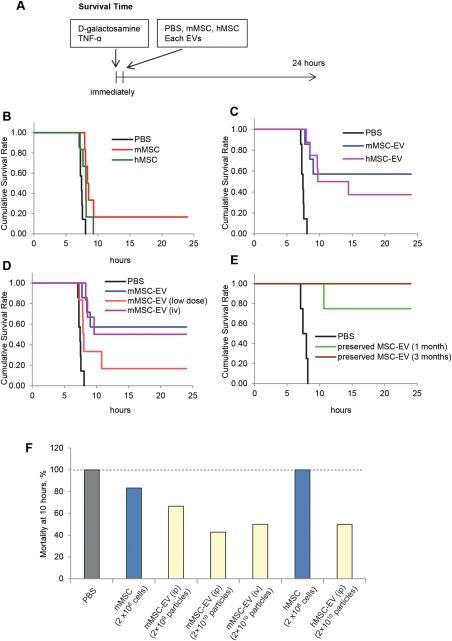
Mesenchymal stem‐cell derived extracellular vesicles (EV) improve survival in experimental fulminant hepatic failure. **(A)**: Hepatic failure was induced in male C57Bl/6 mice by intraperitoneal injection of 20 mg d‐galactosamine/0.3 μg TNF‐α, followed by administration of human MSC, murine MSC, or EV isolated from these cells. Animals were observed for 24 hours. **(B–E)**: Survival at 24 hours in mice following administration of d‐galactosamine/TNF‐α (alone, *n* = 3), or with PBS as a control (*n* = 8) was compared with coadministration of (B) hMSC (*n* = 6) or mMSC (*n* = 6), or (C) hMSC‐EV (*n* = 8), mMSC‐EV (*n* = 7). A dramatic improvement in survival is observed with the use of mMSC‐EV or hMSC‐EV compared with other groups. (D): Survival at 24 hours in mice following administration of d‐galactosamine/TNF‐α and 2 × 10^10^ particles per body mMSC‐EV administered intravenously (*n* = 6) or intraperitoneally (*n* = 7), 2 × 10^9^ particles per body (low dose) mMSC‐EV intraperitoneally (*n* = 6), or PBS (control vehicle) (*n* = 7). (E): Survival at 24 hours in mice following d‐galactosamine/TNF‐α induced hepatic failure with intraperitoneal administration of PBS (*n* = 4), or mMSC‐EVs cryopreserved for 1 month or 3 months (*n* = 4 each) showing enhanced survival benefits of cryopreservation. **(F)**: Comparison of mortality at 10 hours after d‐galactosamine/TNF‐α in different groups. Abbreviations: EV, extracellular vesicles; hMSC, human bone‐marrow derived mesenchymal stem cells; MSC, mesenchymal stem cell; mMSC, murine bone marrow‐derived mesenchymal stem cells; PBS, phosphate‐buffered saline; TNF‐α, tumor necrosis factor‐α.

To evaluate the contribution of MSC‐derived EV (MSC‐EV) to stem cell mediated regenerative effects during liver injury, we first examined the administration of stem cells on survival for 24 hours after receiving d‐galactosamine/TNF‐α. In untreated controls, or in controls receiving PBS (vehicle), the survival was 0% (0 of 7), with most of the deaths occurring within 7–8 hours (Fig. 3B). Administration of human MSC (*n* = 6) did not have any effect on survival, whereas only one of six mice receiving murine MSC survived beyond 10 hours. Next, we examined the effect of EV derived from these cells. mMSC‐EV had a dramatic effect on extending survival compared with 57.1% survival observed at 24 hours. Notably, administration of hMSC‐EV (3/8) also improved survival compared to either untreated or PBS‐treated controls, with 37.5% survival after 24 hours (Fig. 3C). Kaplan‐Meier log rank analysis of survival curves showed a significant extension of survival with either human or mouse MSC or MSC‐EV compared with PBS (Supporting Information Table 1). We also examined the effects of route of administration and dose of mMSC‐EV on survival. A lower mortality was observed following i.p. administration of 2 × 10^10^ particles per body mMSC‐EV compared with 2 × 10^9^ particles per body mMSC‐EV. Survival was similar with either intravenous or i.p. administration of equivalent amounts of mMSC‐EV (Fig. 3D). An analysis of mortality at 10 hours from these studies highlighted the dramatic effect of MSC‐EV in ameliorating TNF‐α/d‐galactosamine induced lethality, with notable beneficial effects observed even across species.

### Cryopreserved MSC‐EV Improve Survival in a Mouse Model of Fulminant Hepatic Failure

To investigate an effect of cryopreservation on therapeutic efficacy of MSC‐EV, we stored mMSC‐EV diluted with PBS at −80°C for 1 to 3 months. Size and concentration were determined using nanoparticle tracking analysis (Table 1). The number of recovered MSC‐EV was reduced at 1 month, but not further reduced at 3 months. The size distribution of recovered EV was similar to that at baseline. The effect of these cryopreserved MSC‐EV on survival from d‐galactosamine/TNF‐α injury was determined. One‐month cryopreserved mMSC‐EV had 75% survival (4/4), whereas 3‐month cryopreserved mMSC‐EV had 100% survival (4/4) compared with PBS controls in which no survival was observed after 24 hours of d‐galactosamine/TNF‐α administration (Fig. 3E).

**Table 1:** Characterization of preserved mesenchymal stem cell‐derived extracellular vesicles

		0 day	1 month	3 months
	Particle count, % of day 0 baseline	100	78.2 ± 1.3	77.2 ± 7.8
EV quantitation	Mean size (nm)	117.3 ± 2.6	118.0 ± 1.0	121.0 ± 2.1
	Modal size (nm)	89.8 ± 2.5	94.7 ± 7.3	90.8 ± 1.6
	% particles <110 nm	62.7 ± 1.2	61.3 ± 0.5	60.0 ± 1.4
Functional effect	% mortality at 9 hours % mortality at 12 hours	50 100	0 25	0 0

MSC‐EV were analyzed after isolation (baseline) or following cryopreservation for 1 month or 3 months. Quantitative analysis of size and number was performed using NanoSight. Functional efficacy was assessed by determining mortality after injection of TNFα/d‐galactosamine. Data represent the mean ± SEM of four separate studies. Abbreviations: EV, extracellular vesicles; MSC, mesenchymal stem cells.

### MSC‐EV Reduce d‐Galactosamine/TNF‐α Induced Hepatic Inflammation and Hepatocyte Injury In Vivo

**Figure 4:**
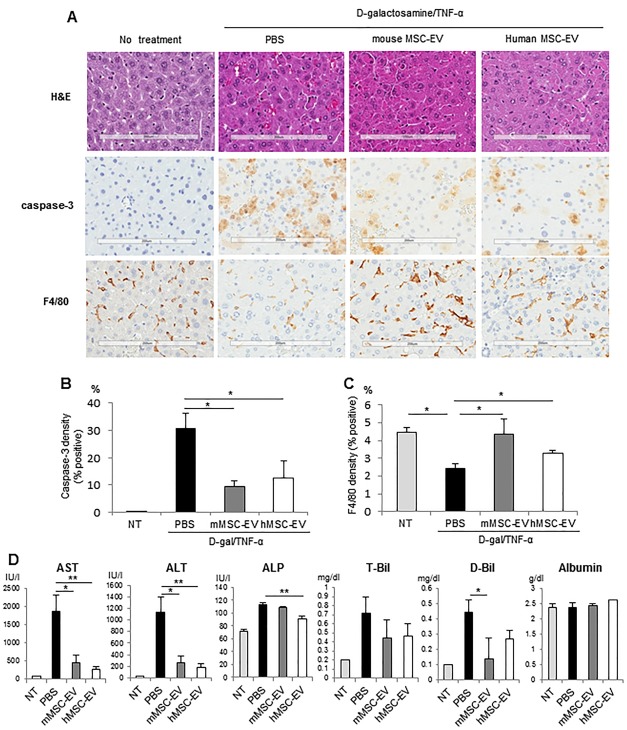
Mesenchymal stem cell‐derived EV reduce d‐galactosamine/TNF‐α induced hepatic inflammation and hepatocyte injury in vivo. To investigate effect of MSC‐EV on hepatocyte injury and inflammation, mice received PBS control (*n* = 6), mMSC‐EV (*n* = 6), or hMSC‐EV (*n* = 6) or no additional intervention (no treatment) immediately after injection of d‐galactosamine/TNF‐α and liver tissues and blood collected after 6 hours. **(A)**: Hematoxylin and eosin activated caspase 3 and F4/80 expression in the liver of mice. Caspase‐3‐positive cells and F4/80‐positive cells are identified by brown staining. Scale bars = 200 μm. **(B, C)**: Whole liver sections were scanned at ultraresolution on the ScanScope XT and positive staining cells were quantified using positive pixel counts by Aperio Imagescope analysis. The percentage of (B) caspase‐3‐positive cells and (C) F4/80‐positive cells are shown for sections from each group. Data represent the mean ± SEM from six livers. **(D)**: Biochemical examination of serum from each group. Data represent the mean ± SEM from six separate animals. *, *p* < .05; **, *p* < .01. Abbreviations: EV, extracellular vesicles; hMSC, human bone‐marrow derived mesenchymal stem cells; MSC, mesenchymal stem cell; mMSC, murine bone marrow‐derived mesenchymal stem cells; NT, nontargeting; PBS, phosphate‐buffered saline; TNF‐α, tumor necrosis factor‐α.

We examined hepatic histomorphology and circulating biochemical markers of hepatic injury 6 hours after i.p. administration of MSC‐EV or PBS (controls) to examine their impact on hepatic injury induced by d‐galactosamine/TNF‐α prior to hepatic failure and death. For these studies, we used mouse MSC‐EV (2 × 10^10^ particles per body) intraperitoneally because this dose of MSC‐EV and route showed the greatest improvement in survival. Histological examination of control mice livers revealed profound hepatocellular apoptosis, hemorrhagic necrosis, enucleated necrosis, and mononuclear leukocyte infiltration with cytoplasmic vacuolization. In contrast, livers from mice treated with either hMSC‐EV or mMSC‐EV had less inflammation (Fig. 4A). Immunohistochemistry for activated caspase‐3 revealed positive cell expression in many cells, and particularly around zone 1 in the PBS group, but a significantly reduced number of caspase‐3 positive cells were observed with either mMSC‐EV or hMSC‐EV (Fig. 4A, 4B). Thus, MSC‐EV reduce hepatic inflammation and hepatocyte apoptosis in vivo following administration of d‐galactosamine/TNF‐α. F4/80 is a marker of macrophage/Kupffer cells, and an increase in F4/80 positive cells was observed following treatment with mMSC‐EV (Fig. 4C). Kupffer cells play a key role in the regulation of hepatic inflammation and hepatocyte death. Their contribution to liver injury is controversial, but their recruitment by MSC‐EV supports a protective role from liver injury in this model.

**Figure 5:**
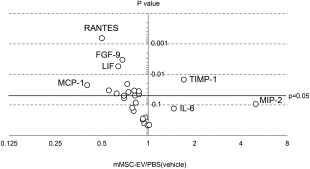
Effect of mesenchymal stem cell derived extracellular vesicles on circulating cytokines and growth factors. The effect of MSC‐EV on serum cytokines or growth factors was determined in serum obtained 6 hours after administration of d‐galactosamine/TNF‐α. The average fold‐change in amount of circulating protein is expressed relative to that in mice receiving PBS as a control, after d‐galactosamine/TNF‐α, and plotted against the *p* value based on data from four animals in each group. Selected differentially altered proteins are labeled. Abbreviations: EV, extracellular vesicles; FGF, fibroblast growth factor; IL, interleukin; LIF, leukemia inhibitory factor; MCP, monocyte chemotactic protein; MIP, macrophage inflammatory protein; mMSC, murine bone marrow‐derived mesenchymal stem cells; PBS, phosphate‐buffered saline; RANTES, Regulated on Activation, Normal T Expressed and Secreted; TIMP, tissue inhibitor of metalloproteinases; TNF‐α, tumor necrosis factor‐α.

Serum cytokines or markers of hepatic injury were analyzed 6 hours after injection of d‐galactosamine/TNF‐α. Compared to PBS controls, both hMSC‐EV and mMSC‐EV decreased mean serum alanine and aspartate aminotransferase level (Fig. 4D). Moreover, a significant reduction in alkaline phosphatase was noted with hMSC‐EV, and in direct bilirubin with mMSC‐EV compared with PBS controls. A suppression of circulating cytokines was observed with mMSC‐EV (Fig. 5), and in particular growth factors such as Epidermal Growth Factor and Stem Cell Factor, and chemokines such as interferon gamma‐induced protein‐10 (IP‐10), Interleukin‐1 α, Macrophage Inflammatory Protein‐3β, Monocyte Chemotactic Protein‐1 and 3 were significantly decreased by mMSC‐EV (Supporting Information Table 2). Although an increase in Tissue Inhibitor of Metalloproteinases 1 and Interleukin‐6 (IL‐6) were observed, the changes observed in the latter were not statistically significant.

### lncRNA Y‐RNA‐1 Is Enriched in hMSC‐EV and Can Protect Hepatocyte from Apoptosis In Vitro

**Figure 6:**
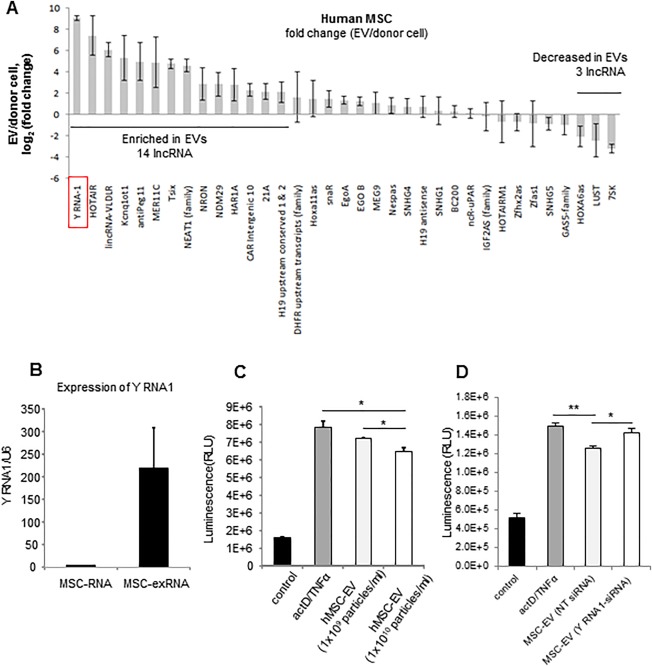
Y‐RNA‐1 is enriched in hMSC‐EV and can reduce TNF‐α/ActD induced hepatocyte apoptosis in vitro. **(A)**: lncRNA expression was assessed by qPCR in hMSC or in EV isolated from these cells. Analysis was performed on four independent samples for each lncRNA. Thirty‐five lncRNAs were identified in hMSC‐EV and hMSC, of which 14 were enriched within EVs. **(B)**: Expression of Y RNA1 in hMSC‐EV and hMSC were independently validated by qPCR in an independent set of three samples each. **(C)**: Caspase 3/7 assay of human hepatocytes (HH) incubated with ActD/TNF‐α in the absence or presence of hMSC‐EV (1 × 10^9^ or 1 × 10^10^ particles per milliliter). Data represent the mean ± SEM of three separate studies. **(D)**: Caspase 3/7 assay of human hepatocytes incubated with ActD/TNF‐α or PBS (control) in the presence or absence of EV‐derived from hMSC transfected with 50 nM siRNA to Y RNA‐1 or NT control. Data represent the mean ± SEM of three separate studies. *, *p* < .05; **, *p* < .01. Abbreviations: ActD, actinomycin D; EV, extracellular vesicles; hMSC, human bone‐marrow derived mesenchymal stem cells; lncRNA, long noncoding RNA; mMSC, murine bone marrow‐derived mesenchymal stem cells; NT, nontargeting; TNF‐α, tumor necrosis factor‐α.

The role of lncRNA in modulating gene expression is being increasingly recognized. We have recently identified selective enrichment of lncRNA within EV released from liver cells, as well as an effect of lncRNA in modulating cell survival in response to hypoxia or chemotherapy in malignant hepatic epithelia. Thus, we postulated that the beneficial effects of MSC‐EV could be mediated through specific lncRNA. To identify such candidate lncRNAs, we first sought to identify lncRNA that enriched within MSC‐EV. Expression profiling was performed using qRT‐PCR‐based assays to identify lncRNA within MSC‐EV. Thirty‐five lncRNAs were detected in MSC‐EV, of which 17 lncRNAs were differentially expressed greater than fourfold in MSC‐EV compared to their expression in donor cells (Fig. 6A). Of these, 14 were enriched while 3 were reduced in expression (Supporting Information Table 3) in MSC‐EV compared to MSC. Among these, the most highly enriched lncRNA in hMSC‐EV was Y‐RNA‐1 (Fig. 6B). Similarly, enrichment of Y‐RNA‐1 was also observed in mMSC‐EV compared with mMSC.

### Y‐RNA‐1 Mediates the Reduction of d‐Galactosamine/TNF‐α Induced Apoptosis by MSC‐EV In Vitro

We next evaluated the effects of MSC‐EV on TNF‐α induced hepatocyte apoptosis in vitro. HH were incubated with 1 × 10^9^ and 1 × 10^10^ particles per milliliter hMSC‐EV or control diluent without EV for 24 hours. Cells were then pretreated with ActD 200 ng/ml, 30 minutes prior to TNF‐α 20 ng/ml. Caspase‐3/7 activity was then assessed after 12 hours. MSC‐EV reduced ActD/TNF‐α induced caspase‐3/7 activity on HH cells in a dose dependent manner, and consistent with a reduction in apoptosis (Fig. 6C). We next examined the effects of Y‐RNA‐1 on hepatocyte apoptosis. EV were isolated from MSC that were transfected with either 50 nM Y‐RNA‐1 siRNA or NT control siRNA. HH were preincubated with these MSC‐EV (1 × 10^10^ particles per milliliter) for 24 hours, prior to incubation with ActD 200 ng/ml per TNF‐α 20 ng/ml. A significant increase in caspase‐3/7 activity was observed in hepatocytes incubated with EV derived from MSC with Y‐RNA‐1 knockdown compared with EV that were derived from MSC transfected with control NT‐siRNA (Fig. 6D). Taken together, these results implicate MSC‐EV Y‐RNA‐1 within MSC‐EV in protection of hepatocyte apoptosis in vitro.

## Discussion

Improvements of liver function in acute hepatic failure that are observed with MSC transfusions have been attributed to their pluripotency and capability to generate functional epithelia. However, even though MSC differentiation into hepatocyte can occur in vitro, it is unclear if complete differentiation occurs in vivo, or even whether or not trans‐differentiation contributes to the therapeutic benefits that are reported with the use of MSC. In contrast, emerging evidence supports the role of paracrine modulators 10 in mediating the therapeutic effects of MSC in acute and chronic liver failure. Soluble factors could have functional trophic and immunomodulatory effects on immune cells responsible for liver injury 10, 13, 28, 29, 30. Systemic injection of MSC‐CM can provide a significant survival benefit in animal models of hepatic failure. Indeed, an increased tendency toward survival was observed with MSC‐CM even though no significant benefit was observed with the infusion of MSC in a rat model of fulminant hepatic failure 31.

MSC‐CM is extremely heterogeneous, and in addition to soluble factors, includes particulate matter such as EV. We postulated that therapeutic effects with the use of MSC‐CM were mediated through EV released from MSC. The subsequent uptake of these EV by recipient cells can result in the transfer of biologically active RNA that can mediate functional effects. Thus, we tested the therapeutic potential of stem cell‐derived EV as a treatment paradigm for acute or fulminant hepatic failure using a murine preclinical model of lethal liver failure. Our findings show that EV isolated from bone marrow‐derived MSC can protect hepatocytes from apoptosis, reduce biochemical and histological hepatic injury, and enhance survival following d‐galactosamine/TNF‐α induced acute hepatic failure in mice. Of note, survival benefits were observed with the use of EV isolated from either human or murine bone marrow derived MSC. In addition to supporting a critical role for MSC‐derived EV in paracrine responses that contribute to reparative responses to hepatic injury, these studies provide compelling evidence to support the use of MSC‐EV as a therapeutic in acute or fulminant hepatic failure.

The preparations of EV that were evaluated in this study are heterogeneous, and may contain distinct types of vesicles with differing biogenesis such as exosomes and microvesicles. While the predominant content is that of a vesicle population with a size consistent with that of exosomes, they may contain other types of vesicles. However, functional effects can be mediated through the transfer of bioactive RNA within different types of vesicles. Recent studies have reported the functional effect of microvesicles derived from human liver stem cell in remnant hepatocytes after hepatectomy 32 and umbilical cord MSC‐exosomes in CCl4‐induced liver fibrosis 33. Responses in recipient cells, such as activation of proliferation or inhibition of epithelial‐mesenchymal transition can be attributed to transfer of RNA. Thus, even though it is not feasible to isolate fully homogenous populations of exosomes, microvesicles, or other EV, the further development of an MSC‐derived EV therapeutic product would not precluded by the heterogeneity of the preparation.

The preclinical model of lethal hepatic failure model is characterized by hepatic injury mediated by inflammatory cytokines. The contribution of EV to modulation of innate immune responses during hepatic injury is unknown. An increase in macrophages within the liver suggests that MSC‐EV are recruiting a protective population of macrophages that contribute to the resolution of inflammation in this model. Consistent with this, there was an increase in macrophage inflammatory protein 2 (MIP2), which is secreted by macrophages and IL‐6, an anti‐inflammatory cytokine with pleiotropic effects. IL‐6 has prominent hepatoprotective effects in liver injury induced by diverse stimuli such as Fas, acetaminophen, Con‐A, LPS/Galactosamine, and carbon tetrachloride 34. IL‐6 is elevated in patients with fulminant hepatic failure and involved in both hepatocyte regeneration and in T‐cell activation and differentiation. In contrast to the observed increase in IL‐6, there was an attenuation of expression of several inflammatory cytokines and chemokines such as IL‐1 alpha, MIP‐3 beta, IP‐10, monocyte chemotactic protein‐1 (MCP‐1), and MCP‐3 observed with mMSC‐EV treatment. These observations suggest that immune responses to injury can be driven by MSC‐EV through modulation of macrophage recruitment or function, or release of anti‐inflammatory mediators.

Y‐RNA‐1 was identified as a noncoding RNA that is highly enriched within both human and murine MSC‐EV. The Y RNAs are a group of noncoding RNAs that have been implicated in cell proliferation and DNA replication through functional interactions with chromatin and initiation proteins such as the origin recognition complex. A model whereby Y RNAs act as cellular stress sensors has also been proposed, to act in the cellular recovery by salvaging misfolded RNAs. Y‐RNA‐1 is upregulated in many human solid tumors such as bladder, cervix, colon, kidney, lung, and prostate adenocarcinomas 35. In vitro, MSC‐EV protected against ActD/TNF‐α induced apoptosis in nonmalignant HH. Although knockdown of human Y‐RNA‐1 has been reported to arrest cell proliferation 36, 37, the effects of Y‐RNAs on modulating apoptosis are not established. In mice, there is a lower expression of Y‐RNA‐1 in the liver, compared to other organs. MSC have been reported to have anti‐apoptotic effects on hepatocytes 4, 5, 6, 7 in vivo. During experimental renal injury induced by cisplatin, MSC‐derived microvesicles were shown to modulate the expression of several different anti‐apoptotic genes or effectors of apoptosis within human tubular epithelial cells 9.

Although our data show that Y‐RNA‐1, enriched within MSC‐EV, may contribute to protection from hepatocyte apoptosis, they do not exclude the possibility that EV may either contain directly cyto‐protective mediators, or factors that modulate apoptosis in recipient hepatocytes. Indeed, the beneficial effect of MSC‐derived EV on reducing mortality in experimentally induced lethal hepatic injury could therefore result from modulation of the inflammatory response as well as activation of protective mechanisms to limit cell death. These possibilities warrant further assessment.

## Conclusion

The observations made herein support a critical role for paracrine effects of MSC‐derived EV in mediating reparative responses following hepatic injury, and provide compelling evidence to support the use of MSC‐derived EV as a therapeutic strategy for fulminant hepatic failure. The demonstration of intrahepatic accumulation of systemically administered MSC‐EV after liver injury, as well as the preservation of functional effects in cryopreserved MSC‐EV further increase the feasibility of their use for therapeutic purposes.

## Author Contributions

H.H. and I.K.Y.: provision of study material or patients; collection and/or assembly of data; data analysis and interpretation; manuscript writing; final approval of manuscript; K.T.: provision of study material or patients; collection and/or assembly of data; data analysis and interpretation; A.M.: data analysis and interpretation; T.P.: conception and design; financial support; administrative support; manuscript writing; final approval of manuscript.

## Disclosure of Potential Conflicts of Interest

The indicate no potential conflicts of interest.

## Supporting information

Supporting Information.
