# Supplementary material for: Extracellular Vesicles from Bone Marrow‐Derived Mesenchymal Stem Cells Improve Survival from Lethal Hepatic Failure in Mice
Source: Stem Cells Transl Med. 2017 Feb 18;6(4):1262–72. doi: 10.1002/sctm.16-0226 (PMC5442843; doi:10.1002/sctm.16-0226)
Supplement: Supplementary file 1 — Supporting Information. [file SCT3-6-1262-s001.docx]

**Supplementary information**

**Extracellular vesicles from bone marrow derived mesenchymal stem cells**

**improve survival from lethal hepatic failure in mice**

Hiroaki Haga, Irene K Yan, Kenji Takahashi, Tushar Patel

Supplementary Table 1. Comparison of survival after TNFα/D-Galactosamine with different treatment interventions.

Supplementary Table 2. Circulating cytokine and growth factors during lethal hepatic injury.

Supplementary Table 3. lncRNA expressed exclusively in extracellular vesicles derived from human mesenchymal stem cells.

**Supplementary Table 1. Comparison of survival after TNFα/D-Galactosamine with different treatment interventions.** Kaplan Meier Log rank analysis of survival at 24 hours for each treatment group is shown.

|  | **PBS** | **mMSC(IP)** | **hMSC(IP)** | **mMSC-EV(IP)** | **mMSC-EV(IP) (low dose)** | **mMSC-EV(IV)** | **hMSC-EV(IP)** |
| --- | --- | --- | --- | --- | --- | --- | --- |
| **PBS** | － |  |  |  |  |  |  |
| **mMSC(IP)** | 0.002 | － |  |  |  |  |  |
| **hMSC(IP)** | 0.014 | 0.153 | － |  |  |  |  |
| **mMSC-EV(IP)** | <0.001 | 0.175 | 0.015 | － |  |  |  |
| **mMSC-EV(IP) (low dose)** | 0.049 | 0.623 | 0.551 | 0.125 | － |  |  |
| **mMSC-EV(IV)** | <0.001 | 0.135 | 0.005 | 0.909 | 0.129 | － |  |
| **hMSC-EV(IP)** | <0.001 | 0.154 | 0.003 | 0.683 | 0.165 | 0.830 | － |

**Supplementary Table 2. Circulating cytokine and growth factors during lethal hepatic injury.** Serum was collected 6 hours after D-Galactosamine/TNF-α and i.p administration of PBS (MSC-EV -) or mMSC-EV (MSC-EV +). Data represents the mean±SEM of analysis from four mice for each group. EGF: Epidermal Growth Factor, FGF: Fibroblast Growth Factor, GM-CSF: Granulocyte-Macrophage Colony-Stimulating Factor, KC/GRO: Growth-Regulated Alpha Protein, IFN: Interferon, IP: Interferon gamma Induced Protein, IL: Interleukin, LIF: Leukemia Inhibitory Factor, M-CSF: Macrophage Colony-Stimulating Factor, MDC: Macrophage-Derived Chemokine, MIP: Macrophage Inflammatory Protein, MCP: Monocyte Chemotactic Protein, OSM: Oncostatin-M, SCF: Stem Cell Factor, RANTES: T-Cell-Specific Protein RANTES, TIMP: Tissue Inhibitor of Metalloproteinases, TNF: Tumor Necrosis Factor, VEGF: Vascular Endothelial Growth Factor , LD: low data.

| Cytokine/Growth Factor | MSC-EV  － | MSC-EV  ＋ |  | p value |
| --- | --- | --- | --- | --- |
| Eotaxin | 944.75±137.93 | 660.00±75.09 | pg/mL | 0.060 |
| EGF | 75.00±4.22 | 51.00±4.12 | pg/mL | 0.003 |
| FGF-9 | 13.75±1.31 | 11.4±0.73 | ng/mL | 0.084 |
| FGF-basic | 17.50±3.50 | 16.5±1.66 | ng/mL | 0.402 |
| GM-CSF | <5.6 | <5.6 | pg/mL | LD |
| KC/GRO | <0.065 | <0.065 | ng/mL | LD |
| IFN-gamma | <191 | <191 | pg/mL | LD |
| IP-10 | 411.00±39.37 | 300.5±16.42 | pg/mL | 0.021 |
| IL-1 alpha | 718.75±49.10 | 588.75±45.00 | pg/mL | 0.049 |
| IL-1 beta | 7.78±0.77 | 7.70±0.23 | ng/mL | 0.464 |
| IL-2 | <93 | <93 | pg/mL | LD |
| IL-3 | <3.0 | <3.0 | pg/mL | LD |
| IL-4 | <227 | <227 | pg/mL | LD |
| IL-5 | <0.75 | <0.75 | ng/mL | LD |
| IL-6 | 29.00±5.57 | 42.5±8.39 | pg/mL | 0.084 |
| IL-7 | <0.45 | <0.45 | ng/mL | LD |
| IL-10 | 294.30±38.68 | 236.50±30.36 | pg/mL | 0.158 |
| IL-11 | <152 | <152 | pg/mL | LD |
| IL-12p70 | <0.18 | <0.18 | ng/mL | LD |
| IL-17A | <0.015 | <0.015 | ng/mL | LD |
| IL-18 | 41.00±2.52 | 39.25±0.48 | ng/mL | 0.260 |
| LIF | 1667.50±159.24 | 1065.75±44.81 | pg/mL | 0.005 |
| M-CSF-1 | 10.43±0.53 | 9.1±0.39 | ng/mL | 0.045 |
| MDC | 2477.50±436.72 | 1545.00±111.77 | pg/mL | 0.042 |
| MIP-1 alpha | 5.95±0.68 | 5.48±0.53 | ng/mL | 0.301 |
| MIP-1 beta | 1797.50±137.14 | 1416.75±263.49 | pg/mL | 0.124 |
| MIP-2 | 63.75±6.09 | 318.00±170.63 | pg/mL | 0.094 |
| MIP-3 beta | 5.18±0.56 | 3.85±0.26 | ng/mL | 0.038 |
| MCP-1 | 3140.00±697.84 | 1260.75±261.58 | pg/mL | 0.023 |
| MCP-3 | 2002.50±352.15 | 1110.50±187.52 | pg/mL | 0.033 |
| MCP-5 | 95.00±14.47 | 66.00±3.44 | pg/mL | 0.050 |
| OSM | 0.65±0.08 | 0.60±0.02 | ng/mL | 0.284 |
| SCF | 2337.50±87.21 | 2030.00±110.08 | pg/mL | 0.036 |
| RANTES | 0.16±0.01 | 0.08±0.01 | pg/mL | 0.001 |
| Thrombopoietin | 86.25±7.03 | 70.00±2.00 | ng/mL | 0.034 |
| TIMP-1 | 6.63±0.82 | 11.28±1.42 | ng/mL | 0.015 |
| TNF-alpha | <0.11 | <0.11 | ng/mL | LD |
| VEGF-A | 265.75±24.78 | 269.75±22.85 | pg/mL | 0.455 |

**Supplementary Table 3. lncRNA expressed exclusively in extracellular vesicles derived from human mesenchymal stem cells.**

| lncRNA | |  | Absolute fold change | |
| --- | --- | --- | --- | --- |
| Y RNA-1 | |  | 535.6 |  |
| HOTAIR | |  | 172.4 |  |
| lincRNA-VLDLR | |  | 67.8 |  |
| Kcnq1ot1 | |  | 40.3 |  |
| antiPeg11 | |  | 31.7 |  |
| MER11C | |  | 30.0 |  |
| Tsix | |  | 27.2 |  |
| NEAT1 (family) | |  | 24.3 |  |
| NRON | |  | 7.5 |  |
| NDM29 | |  | 7.2 |  |
| HAR1A | |  | 7.0 |  |
| CAR Intergenic 10 | |  | 5.0 |  |
| 21A | |  | 4.5 |  |
| H19 upstream conserved 1& 2 |  | | 4.3 |  |
